# Monitoring of Cutting Process and Tool Condition of Metal and Metal Composite

**DOI:** 10.3390/ma16103660

**Published:** 2023-05-11

**Authors:** Paweł Twardowski, Michał Wieczorowski

**Affiliations:** Faculty of Mechanical Engineering, Institute of Mechanical Technology, Poznan University of Technology, Piotrowo Street 3, 60-965 Poznan, Poland; michal.wieczorowski@put.poznan.pl

Machining is a manufacturing process that involves the use of machines to remove materials from a workpiece to create a desired shape and size. The process can be used to produce a wide range of components and parts, from simple bolts and screws to very complex elements. The use of machining is widespread in various industries, including automotive [1], aerospace [2], defense [3], medical [4,5], electronics [6], and many others [7]. Machining is particularly important in the machine industry, where precision and accuracy are critical to the proper functioning of machines and equipment. Machining also allows for customization and flexibility in the production of parts, enabling manufacturers to meet the specific needs of their customers.

Machine parts with high precision must be obtained and employed to ensure that machines and equipment operate smoothly and efficiently. Precision machining involves the use of advanced machinery and equipment to achieve tight tolerances and high accuracy [8]. This requires skilled technicians and engineers who can operate and program the machines to produce the desired results. Precision machining plays a critical role in many applications, including those where safety and reliability are paramount (aerospace or defense). Machined components are also used in the medical industry, where they must meet strict regulatory requirements and ensure the safety of patients. Sometimes surfaces produced must have specific surface topography, which may require two or more processes for manufacturing a finished object [9].

The topic of machining has been known for many years but is nevertheless still present in the scientific literature in this field. It concerns many aspects related to the manufacturing process, among which tool wear processes play a leading role [10,11]. The wear on different tool surfaces is studied [12], depending on a number of factors. The machining strategy is also important [13], as it influences functional properties, usability, and surface topography. Furthermore, the choice of cutting conditions [14] is an important issue that determines the functioning of elements and the whole assembly set.

While steel and cast iron have traditionally been the most commonly used construction materials in machining, advancements in technology have introduced more advanced and difficult-to-cut materials into the market, such as composites [15,16]. These new materials offer superior strength and durability, making them highly desirable in industries where strength and durability are essential. Composites are made up of two or more different materials, such as fibers and resins, that are combined to form a stronger material. They are often used in the aerospace and defense industries due to their high strength-to-weight ratio and resistance to extreme temperatures and environments. However, the use of these advanced materials in machining has posed significant challenges. Composites, for example, are known to cause tool wear and delamination during machining, making it difficult to achieve precise cuts and finishes. As a result, machining composite materials requires specialized equipment and techniques and highly skilled operators who understand the properties of the materials being machined. This is due to many phenomena, including minimum chip thickness [17], machining settings [18], and cutting force [19,20]. The modeling of force also plays an important role [21,22], being a very useful prediction tool.

The shrinking use of traditional materials such as steel and cast iron has led to the development of new machining techniques and equipment that can effectively handle these difficult-to-cut materials. Manufacturers are investing in new cutting tools and technologies, such as laser cutting and waterjet cutting, that can cut composites and other advanced materials with greater ease and precision.

In the cutting process, there are many important factors influencing its characteristics as well as the surfaces that are obtained as a result. The Special Issue—*Monitoring of Cutting Process and Tool Condition of Metal and Metal Composite*—included articles on the topics related to this, which shows, on the one hand, how wide the scope of the research being conducted is and, on the other hand, how interconnected the different processes are and how they determine the functional behavior of finished surfaces and—in turn—the entire objects.

Starting from classical machining, Tabaszewski, Twardowski, et al. [23] compared different intelligent system methods to identify the tool wear during this kind of machining. They made their tests on gray cast iron using carbide cutting inserts. An experimental investigation was conducted with various cutting speeds to determine the exact value of depth of cut and feed rate. Furthermore, based on the vibration acceleration signals, measures were developed that were correlated with the tool condition. In the study, selected methods (classification and regression tree, induced fuzzy rules, and artificial neural network) were evaluated to find the most effective model. For milling, Wang, Zhang, and Li [24] presented an experimental study on the effect of processing parameters on milling forces and surface topography for conventional milling and longitudinal-torsional ultrasonic vibration milling of the Al–Li alloy. The variable depth of cut and tool chip pulling were the advantages of ultrasonic machining. The high-frequency impact generated by the longitudinal vibration not only reduces the chip accumulation on the surface but also smooths out the tool-tooth scratches and creates a regular surface profile. In addition, the analysis of the spectrum of the collected milling forces revealed that the ultrasonic vibration caused the high-frequency components of the milling forces. The results proved the possibilities and usefulness of ultrasonic machining. Drilling in difficult-to-cut material was investigated by Bronis, Miko, et al. [25], who discussed experimental results concerning the quality of through holes drilled in Inconel 718. The tests involved different values of the feed per revolution and spindle speed, as well as different types of kinematic systems. Three kinematic systems were considered: based on the driven tool holder, based on the spindle rotations, and when the workpiece and the tool rotated in opposite directions (this option proved to be the best). The findings confirm that the kinematic system, as well as the feed per revolution, are the key factors affecting the quality of holes drilled in Inconel 718.

Let us move now to grinding and finishing. Kacalak, Lipinski et al. [26] discussed the technological aspects of the diagnostics of grinding processes, including the main features of the grinding process and their importance in diagnostic issues. The authors pointed out that the parameters used to assess the topography of the ground surfaces do not have sufficient possibilities to differentiate the surface condition of the grinding wheels. New dedicated parameters to assess the properties of the grinding wheel surface were proposed. These parameters have a high ability to differentiate changes occurring as a result of the abrasion of grain vertices, their chipping, or the loading of the grinding wheel surface. The methodology for assessing the processes of abrasive grain wear and changes in the shape and dimensions of the grinding wheel, taking into account the probabilistic features of the grinding process, was formulated. An idea of the use of additive technology to produce specialized abrasive tools, including those with built-in process sensors was also presented. Grinding was also a matter of research performed by Hamrol, Hoffmann, et al. [27]. They studied belt grinding, commonly used in the finishing of non-functionally shaped surfaces of surgical instruments. They considered the possibility of replacing manual belt grinding with a robotic process, taking the example of the arms of orthodontic forceps. The condition of the treated surface, defined by its structure and roughness, and the geometric accuracy and error of the shape of the arm in the selected cross-section were adopted as the comparative criteria. Research has shown that robotic belt grinding is more efficient in terms of quality and produces more consistent results than manual grinding. In finishing, Juniewicz, Plichta, et al. [28] presented the results of experimental studies of the centrifugal disc finishing (CDF) process of steel elements with the use of an active workpiece holder that allows workpieces for additional rotational and oscillational movements. The mechanism of formation of the surface texture was evaluated, and the intensity and effectiveness of the machining process were assessed. It was found out that additional movements of the workpiece significantly affect the formation of the machining traces generated by the elementary phenomena of micro-cutting, scratching, grooving, etc. These combined and complex interactions, as a result, lead to the formation of the surface topography of the workpieces. The presented research results show that the use of an active holder, enabling rotation and oscillation of the workpiece, may lead to a more effective use of smoothing processes in CDF machines. A similar topic was shown by Skoczylas and Zaleski [29], who investigated the impact of finishing methods on surface topography, roughness, microhardness of the surface layer, residual stresses, and fatigue life of workpieces made of C45 steel. The samples were prepared by slide burnishing, ball burnishing, centrifugal shot peening, centrifugal shot peening + slide burnishing, and centrifugal shot peening + ball burnishing. It was noted that, after finishing, the surface roughness parameters decreased significantly in relation to the reference surface, with the exception of the centrifugal shot peening technology. The highest increase in microhardness was obtained after centrifugal shot peening and slide burnishing, while the combination of centrifugal shot peening and ball burnishing resulted in the highest absolute value of compressive residual stresses.

Brushing to eliminate defects is somehow related to the above-mentioned finishing operations. Here, Matuszak, Zaleski, et al. [30] showed the results of a study on the effectiveness of removing surface defects by brushing, as damage to machine components usually begins on their surface or in the surface layer area. Experiments were made in which surface defects were generated on a specially designed test stand, where it was possible to affect their geometry, depth, and width. It was shown that under certain conditions of brushing treatment, surface defects could be effectively removed. The results demonstrated that brushing was an effective method for strengthening the surface layer and that the value of strengthening in the area of defects depended on the effectiveness of their removal.

Another topic discussed in the issue was manufacturing thin walls. Ciecielag and Zaleski [31] analyzed the face milling of thin-walled elements made of titanium alloy, aluminum alloy, and polymer composite used in the aviation industry. These materials were milled with folding double-edge cutters with diamond inserts. Maximum vertical forces and surface roughness obtained after machining elements of different thicknesses and unsupported element lengths were investigated with the results of the deformation of milled elements. Zawada-Michałowska, Kuczmaszewski, and Piesko [32], on the other hand, analyzed the effect of the selected geometric properties of thin-walled structures on post-machining deformations. Based on the results of measurements, it was found that absolute deformation values were higher for walls arranged in a semi-open structure. It is related to the lower rigidity of the tested structure resulting from the lack of a stiffening wall, which is the so-called “rib”. As an outcome, the use of high-speed cutting (HSC) provided positive outcomes in terms of minimizing the deformation of thin-walled elements. 

It goes without saying how important temperature issues are in processing. In their research, Cui, Wang, et al. [33] continuously observed high temperatures in the tool-chip contact during the titanium alloy milling process as a factor that affects the tool life and precision of machining. A finite element simulation model of the milling process was established to obtain the highest temperature location in the tool-chip contact area. They created a special temperature sensor using thin film depositing. The measurement results showed that the sensor can monitor the transient temperature in the tool-chip contact area, and its temperature measurement performance showed no detrimental effect from wear. Zawada-Tomkiewicz, Tomkiewicz, and Pela [34] considered measurements in a slightly wider way. They described an automatic system for measuring and compensating for errors resulting from the cutting process in order to improve the accuracy parameters of the workpiece. The measured features were the diameter of the workpiece at two points and the temperature at the end face of the workpiece. Based on the measured values, the process stability was checked, and an error correction value was determined for the next item. It was found that the Autoregressive with Extra Input (ARX) model and the Nonlinear Autoregressive with Extra Input (NLARX) model, with a neural network, are able to map the inertia of the system and map the process with reasonable accuracy parameters. The research shows that the temperature compensation model is nonlinear and that the best accuracy parameters of the workpiece can be achieved thanks to repeatable measurement and compensation techniques.

Even from this brief description, it is clear how important it is to properly monitor the whole cutting process as well as tool condition when machining metal and metal composites. Machining composite materials presents unique challenges, particularly when it comes to tool life and surface roughness [35,36]. Unlike conventional materials such as steel and cast iron, composite materials have a much shorter tool life due to their abrasive nature. Additionally, the machining process can also have a significant impact on the surface roughness and other technological effects of the finished product. To address these challenges, monitoring systems have been developed that can assess the machining process and tool life in real-time. These systems use signals such as acoustic emission, cutting forces, vibrations, noise, or temperature [37] to extract appropriate features and identify the process and tool state. By monitoring these signals, the system can detect changes in the machining process and adjust accordingly to ensure that the tool life is maximized and the surface roughness is within acceptable limits. The use of monitoring systems has the potential to significantly improve the technological effects and process efficiency of machining composite materials. By providing real-time feedback on the machining process, operators can make adjustments on the fly, reducing the risk of tool breakage and minimizing scrap rates. This, in turn, can lead to significant cost savings and improved product quality. Moreover, the random factor that is relevant during surface layer formation of composite materials can be addressed with the help of monitoring systems. These systems can detect variations in the machining process that can affect the surface roughness and other technological effects of the finished product. By making adjustments based on real-time feedback, the system can ensure that the surface roughness and other characteristics are consistent across all parts produced, regardless of the random factor [38].

Surface topography plays a critical role in the machining of construction materials [39,40]. It can affect mechanical properties as well as aesthetic appeal and functionality. Therefore, it is essential to understand the factors that influence surface topography and how to optimize the machining process to achieve the desired results. The surface topography of a machined part is influenced by a variety of factors, including the cutting parameters, tool geometry, material properties, and machining conditions. In particular, the abrasive nature of composite materials can result in high tool wear and deformation, which can cause surface irregularities and defects. As a result, the machining process must be carefully controlled to minimize these effects and achieve the desired surface finish. A proper choice of parameters to characterize surface topography is also very important in this case [41]. It is particularly important for machined surfaces with functional properties [42].

There are several techniques available for measuring the surface topography of composite materials [43]. Contact profilometry involves scanning a stylus over the surface of the composite material and measuring the height variations. The stylus is usually a diamond-tipped probe that is moved over the surface in a controlled manner, and the height variations are measured by a displacement sensor. Contact profilometry provides high-resolution surface measurements but can potentially damage the surface of the material. For this purpose, replicas can be used in some cases [44]. Optical profilometry uses optical sensors to measure the surface topography of composite materials [45,46]. The sensors can be based on a variety of principles, such as confocal microscopy, interferometry, or focus variation. Optical profilometry also provides high-resolution surface measurements without damaging the surface of the material. For some more demanding applications, atomic force microscopy (AFM) or scanning electron microscopy (SEM) can be used. AFM is based on scanning a sharp probe over the surface of the composite material and measuring the forces between the probe and the surface. SEM involves scanning the surface of the composite material with a focused electron beam and detecting the secondary electrons emitted from the surface. In addition to high-resolution surface measurements, AFM and SEM also provide information about surface chemistry and morphology. A technique for composite surface inspection is X-ray computed tomography (CT). This technique involves scanning the composite material with X-rays and using computer algorithms to reconstruct a three-dimensional image of the surface topography. X-ray CT is non-destructive but has limited resolution for surface measurements, though it can visualize re-entrant surfaces [47].

In conclusion, there are several techniques available for measuring the surface topography of composite materials, each with its advantages and limitations. The choice of technique depends on factors such as the required resolution, the size and shape of the material, and the required surface information.
